# The Underlying Mechanisms of Comorbid Anxiety and Depression Among Young Women: Evidence From Brain Structure and Hormone

**DOI:** 10.1155/da/9917994

**Published:** 2025-09-08

**Authors:** Yao Meng, Zhuoling Li, Lulu Hou, Yan Ji

**Affiliations:** ^1^School of Nursing, Nanjing Medical University, Nanjing, Jiangsu, China; ^2^School of Psychology, Shanghai Normal University, Shanghai, China

**Keywords:** brain structure, comorbid anxiety and depression, depressive symptoms, hormone, progesterone, young women

## Abstract

**Aim:** Youth is a time of a significant rise in depressive symptoms, particularly impacted by anxiety in females. However, the identification of the transition from anxiety to depression in young women remains ambiguous. This study collects data on brain structure and hormone levels in young women, intending to investigate the neurophysiological differences among women with anxiety disorders and comorbid anxiety and depression (CAD).

**Methods:** 53 young women were divided into three groups, namely an anxiety group, a CAD group, and a control group, aiming to explore the differences in brain structure and ovarian hormone levels.

**Results:** The CAD group exhibited significantly reduced gray matter volume (GMV) in the right superior frontal gyrus (SFG; 0.38 ± 0.05) and right middle occipital gyrus (MOG; 0.37 ± 0.04) compared to the anxiety (SFG: 0.41 ± 0.04; MOG: 0.41 ± 0.04) and control groups (SFG: 0.45 ± 0.04; MOG: 0.44 ± 0.03; all *p*  < 0.001). Cortical thickness in the right SFG was also significantly lower in the CAD group (2.81 ± 0.24) than in the anxiety (3.08 ± 0.21) and control groups (3.11 ± 0.19; *p*  < 0.001). Progesterone was negatively correlated with GMV in the right MOG (*r* = −0.48, *p*=0.042) and SFG (*r* = −0.53, *p*=0.020) only in the CAD group. Further, no significant associations were observed between estradiol levels and brain structure, nor between anxiety/depression scores and hormone or brain data (all *p*  > 0.070).

**Conclusions:** The change of brain structure in the SFG and MOG may be one of the mechanisms underlying the progression of symptoms from anxiety to CAD, which may also be related to the increase in progesterone, indicating the exacerbation of emotional disorders in young women.

## 1. Introduction

Depression is a common neuropsychological disorder today and is also the main cause of disability worldwide. Women are disproportionately affected, with global estimates that depression affects 4.1% of women compared with 2.7% of men [1]. Studies have shown that the gender difference in depression is first observable during the early adolescent period and becomes pronounced by late adolescence [2]. Depression increases much more substantially in girls, to about twice that of boys [3, 4]. Further, studies reveal that from early adulthood to old age, women are more likely than males to suffer from depressive illnesses [5–7]. It can be said that youth is a period of significant increase in depression in women and a turning point in the surge of depressive symptoms [8]. It is essential to investigate the underlying causes and internal mechanisms of depressive symptoms in young women.

Anxiety is an important risk factor affecting depression [9, 10]. Research shows that most young individuals with depression initially display noticeable anxiety symptoms [11]. Over time, typically ranging from 1 month to a year or longer, depressive symptoms develop, leading to what is known as comorbid anxiety and depression (CAD) [12]. Further study has found that depression in women is caused by long-term anxiety that cannot be alleviated. Women-specific stressful events such as menstruation, childbirth, and menopause, if not properly resolved, can lead to prolonged periods of stress, ultimately resulting in depression [13]. Therefore, the shift from anxiety to depression is common in clinics and is important for understanding how depression begins. Clarifying the differences between individuals with anxiety and those with co-occurring depression and anxiety can help to identify high-risk populations early and develop better treatment strategies.

The neurophysiological differences are essential for uncovering the underlying causes of individual emotional disorders. Structural and functional neuroimaging studies have demonstrated that depressed patients exhibit various alterations in brain regions involved in anhedonia and emotional processing, such as the anterior cingulate gyrus, frontal lobe, insula, superior temporal gyrus (STG), and basal ganglia [14]. Meanwhile, abnormal brain areas associated with anxiety include the amygdala, anterior cingulate gyrus, and insula [15–17]. These findings evidence some overlap in altered brain regions in depression and anxiety, especially the cortical limbic involving the temporal and orbitofrontal lobes, suggesting that damage to limbic system neural circuits may be a mechanism of CAD. Further, some studies have shown that the cortical gray matter volume (GMV) in individuals with CAD has significantly reduced in the superior frontal gyrus (SFG) and superior parietal gyrus (SPG) [18–20], and the decrease in the volume of the prefrontal cortex in patients is related to the severity of depressive symptoms. However, studies demonstrated that the increase in GMV happened in social anxiety disorder (SAD) patients, which may be the result of continuing efforts to cope with or try to regulate emotions [20]. Besides, a longitudinal study confirmed that the severity of depressive symptoms was associated with a thinner anterior cingulate cortex (ACC) [21]. Some researchers also believed that cortical thickness patterns were distinct between bipolar depression and MDD, but there was a decrease in both patients [22]. In a word, the GMV and cortical thickness in the limbic system are distinct in patients with depression and anxiety. This difference may be the primary factor in elucidating the distinction between women with CAD and those with anxiety disorders unaccompanied by depression.

In addition, studies measuring hormones have shown that decreased levels of ovarian steroid hormones (e.g., estrogen and progesterone) in women of childbearing age are closely related to the development of negative emotions [23–25]. The decrease of estrogen and progesterone is accompanied by changes in the concentration and activity of neurotransmitters, such as 5-hydroxytryptamine (5-HT) and gamma-aminobutyric acid (GABA), which can lead to negative emotions [26]. However, further research suggests that the appropriate secretion and regulation of ovarian hormones can enhance neuroplasticity in brain regions such as the frontal lobe, ACC, and hippocampus, leading to alterations in brain structure and, consequently, helping to alleviate negative emotions in women [27, 28]. In other words, healthy hormone function can inhibit the occurrence of mood disorders by enhancing brain plasticity. Even so, current research consistently indicates that abnormal secretion of ovarian hormones is closely associated with the occurrence of depression in women, but its relationships with anxiety remain unclear: some studies have found that steroid hormones may help alleviate anxiety, while others suggest they may worsen it [29, 30]. Therefore, we speculate that, compared to healthy women, those with CAD have abnormal hormone levels associated with significant brain structural differences, whereas women with anxiety may not exhibit distinct brain structural changes. However, direct evidence linking hormone levels to brain structural changes in CAD patients is still lacking, and our study aims to bridge this gap by empirically examining the relationship between hormone levels and brain structural alterations in women with CAD and anxiety.

Therefore, three groups of young women, the anxiety group, the anxiety–depression comorbidity group, and the control group, were selected as the research objects in this study by analyzing the data of the brain structure and hormone. The study proposed the following hypothesis: (1) compared with the anxiety and control group, the volume of the SFG and the SPG decreased in the subjects with anxiety–depression comorbidity group; (2) compared with the anxiety and control group, the cortical thickness of the limbic system became thinner in the subjects with anxiety–depression comorbidity group; and (3) compared with the anxiety and control group, the levels of estrogen and progesterone in the subjects with anxiety–depression comorbidity group were lower, and the levels were significant related to the changes of GMV and thickness.

## 2. Methods

### 2.1. Participates

Participants were recruited from two local universities and through an advertisement on a website. First, 304 women were invited to fill out the basic personal information and questionnaires of the Beck Depression Inventory (BDI) and the Beck Anxiety Inventory (BAI). The exclusion criteria for participants were met: (1) previous diagnosis of mental illness or use of psychotropic medication; (2) irregular sleep; (3) alcohol and other drug use (i.e., hormonal compounds, oral contraceptives, or psychotropic drugs); and (4) unsuitable for MEG and MRI scans. Second, severe anxiety disorder is linked to a higher risk of later depression [31]. Therefore, this study included individuals with high anxiety (BAI >26) in both an anxiety group and an anxiety–depression comorbidity group. According to the clinical cutoff values, 58 women (mean age = 21.34 ± 2.10) were finally recruited to participate in this study. We assigned those with BAI scores >26, and BDI scores <14 into the high anxiety group (*n* = 19), while those with BAI scores >26, and BDI scores >14 into the anxiety–depression comorbidity group (*n* = 18), those with BAI scores <26, and BDI scores <4 into the control group (*n* = 21). 58 participants were split into two hospitals for MRI scanning because of the partner hospital arrangement. Both hospitals use 3.0T MRI scanners. After completing the segment in VBM, five subjects with IQR scores lower than 80 in the result file report were removed. In the end, there were 17 participants in the high anxiety group, 16 participants in the anxiety–depression comorbidity group, and 20 participants in the control group.

Further, due to research needs, saliva samples from women are collected for hormone level analysis. However, hormone levels are not only influenced by emotions but also by the menstrual cycle of women. Therefore, in order to eliminate the interference of the cycle on women, we selected women with regular periods and collected saliva uniformly during their premenstrual phase (expected within a week before menstruation). A regular menstrual cycle was defined as lasting 21–35 days, with fluctuations within 7 days, and bleeding lasting 3–7 days.

This study was approved by the Ethical Evaluation of Research Projects at the Department of Psychology in the School of Social and Behavioral Sciences at Nanjing University. All participants provided written informed consent, and each was paid 150 yuan for participation. All procedures contributing to this work were performed following the Declaration of Helsinki 1975, as revised in 2008.

### 2.2. Materials

#### 2.2.1. BDI

The Chinese version of the BDI was used to assess individual depression symptoms. The scale consists of 21 items, each rated on a 4-point scale from 0 (no) to 3 (extremely heavy), reflecting respondents' affective state. Recommended guidelines suggest scores of 0–13 indicate minimal or no depression, 14–19 mild depression, 20–28 moderate depression, and 29 or above severe depression [32]. The scale demonstrated acceptable reliability in this study (Cronbach's *α* = 0.88).

#### 2.2.2. BAI

The Chinese version of the BAI was used to assess individual anxiety symptoms. The scale consists of 21 items, rated on a 4-point scale from 1 (no) to 4 (extremely heavy), reflecting respondents' affective state. The BAI used a standard score, obtained by *Y* = int (1.19*X*), where X represents raw scores. Recommended guidelines suggest scores of 0–7 indicate minimal anxiety, 8–15 mild anxiety, 16–25 moderate anxiety, and 26–63 severe anxiety [33]. The scale demonstrated acceptable reliability in this study (Cronbach's *α* = 0.85).

#### 2.2.3. Hormone Collection and Assays

Participants were asked to refrain from consuming high-fat and high-protein foods the day before sample collection, as well as avoid alcohol intake. They were also required to abstain from food and water consumption for half an hour before the sampling procedure. All saliva samples were collected using a Cayman sampling device and stored at −20°C until they were analyzed. Competitive enzyme-linked immunosorbent assay (c-ELISA), a validated and widely used method, was employed to measure estradiol and progesterone levels in females [34]. The estradiol assay had a detection range of 1–100 pg/mL with a sensitivity of 0.6 pg/mL, while the progesterone assay had a range of 10–5000 pg/mL and a sensitivity of 5 pg/mL. The intra-assay and inter-assay coefficients of variation for these analyses were less than 12%.

### 2.3. Experimental Procedure

After entering the lab, participants signed the informed consent form and completed the questionnaires about their levels of depression and anxiety. Then, they received instructions and familiarized themselves with the scanning procedure. After the experiment, saliva was collected to analyze participants' hormone levels (estradiol and progesterone).

### 2.4. MRI Data Acquisition

Half participants' images were acquired using a 3-T GE MRI scanner. A T1-weighted image used a magnetization-prepared rapid acquisition gradient echo (MPRAGE) with the following parameters: repetition time (*T*_R_) = 8.21 ms, echo time (*T*_E_) = 3.18 ms, flip angle = 15°, slice thickness = 1.0 mm, matrix size = 256 × 256, voxel size = 1 × 1 × 1 mm^3^. Each run consisted of 148 slices.

Another half participants' MRI data were also acquired through a 3-T GE MRI scanner. It was used to record images of the high-resolution T1-weighted image using a similar MPRAGE sequence: repetition time (*T*_R_) = 6.68 ms, echo time (*T*_E_) = 2.93 ms, flip angle = 12°, slice thickness = 1.0 mm, matrix size = 256 × 256, voxel size = 1 × 1 × 1 mm^3^. Each run consisted of 192 slices.

### 2.5. Statistical Analysis

#### 2.5.1. Preprocessing of MRI Data

First, SPM12 and CAT12 were used to preprocess the data to obtain brain volume data, the main steps included: (1) the local adaptive segmentation algorithm was used to complete the image segmentation of gray matter, white matter and cerebrospinal fluid tissue in individual space; (2) the geodesic shooting algorithm was used to register participants' brain tissue images from the individual space to the Montreal Neurological Institute (MNI) 152 standard space, and the spatial resolution of the brain tissue images was resampled to 1.5 mm × 1.5 mm × 1.5 mm; (3) the gray matter density was modulated using the Jacobin determinant generated during spatial normalization and the change in gray matter density caused by spatial normalization were removed; and (4) the density maps of GMV after Jacobin modulation was smoothed to reduce spatial noise with a 8 mm Gaussian kernel.

Second, the FreeSurfer 7.3.2 image analysis suite (http://surfer.nmr.mgh.harvard.edu/) was used to obtain the cortical data. The processes are as follows: first, the data from all participants were processed using the fully automated FreeSurfer “recon-all” standard procedure, which included nonuniform intensity correction, skull stripping, Talairach transformations, normalization and atlas registration, subcortical segmentation, surface reconstruction, cortical atlas registration and segmentation, and other processes. Then, the cortical thickness was calculated according to the freesurfer preprocessing data, and the smooth kernel of 10 mm was used for smoothing.

Finally, the ComBat technique (https://github.com/Jfortin1/ComBatHarmonization) was used to harmonize the data [35]. The ComBat technique has been validated in MRI for the harmonization of cortical thickness measurements across scanners [36]. Later, Orlhac et al. [37] further proved that ComBat harmonic can effectively eliminate cross-center technical inconsistencies in radiomics feature values and increase the sensitivity of studies using data from multiple scanners. Recently, Ma et al. [38] also used this method in the multicenter structural MRI. Therefore, the use of the ComBat technique in this study would effectively reduce scanner effects and improve statistical testing power.

#### 2.5.2. Data Analysis

For statistical analysis of GMV data, we established a general linear model in SPM12 with BMI, age, and intracranial total volume (TIV) as covariates. In addition, in order to reduce the influence of nongray matter areas, the statistical range was limited to voxels with GMV > 0.2. AlphaSim correction was employed to control for multiple comparisons, aiming for a corrected *p*-value of less than 0.05, as determined by Monte Carlo simulations. This approach utilized a voxel-wise threshold of *p*  < 0.005 in combination with a minimum cluster size calculated using the AlphaSim program integrated within DPABI. (https://afni.nimh.nih.gov/pub/dist/doc/manual/AlphaSim.pdf).

For the statistical analysis of cortical thickness, we used the query design estimate contrast (Qdec) application embedded in the FreeSurfer program to establish a general linear model, and age and BMI were used as covariates. The results were corrected for multiple comparisons with a cluster-wise level of *p*  < 0.05, and a vertex-wise level of *p*  < 0.005 using the Monte Carlo simulation. Then, the brain regions with significant group differences in GMV and cortical thickness were extracted for ANOVA to further identify the between-group differences, and post hoc multiple-comparisons were corrected by Bonferroni.

Finally, after preliminary testing revealed a skewed distribution in the hormone data, Spearman correlation was applied in the final analysis to examine the relationship between hormone levels and MRI data within each group.

## 3. Results

### 3.1. Demographic Variables

The results of the ANOVA indicate no significant differences in age, BMI, and estradiol and progesterone among the three groups. The BAI and BDI scores of pairwise comparison among the three groups were significantly different (all *p*  < 0.001). Descriptive statistics for the demographic variables, questionnaires, and hormones of the three groups are listed in Table 1.

### 3.2. GMV Results

**Figure 1:**
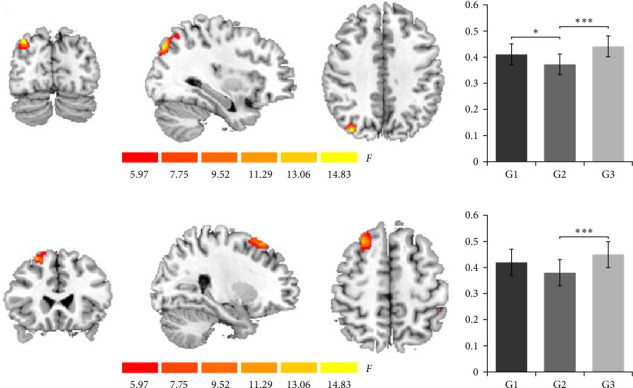
There were significant differences in the GMV of the (a) right middle occipital gyrus and (c) the right superior frontal gyrus among the three groups, and the GMV of the three groups in the corresponding brain regions (the right middle occipital gyrus and right superior frontal gyrus are shown in (b) and (d), respectively). *Note:* G1 = anxiety group; G2 = anxiety–depression comorbidity group; G3 = control group. *⁣*^*∗*^ indicates *p*=0.031, while *⁣*^*∗∗∗*^ indicates *p* < 0.001.

The results showed that there were significant differences in the GMV of the right middle occipital gyrus (MOG, [33−79.5–40.5], *k* = 566, *F* = 16.60; Figure 1a) and the right SFG ([25.5–24–55.5], *k* = 536, *F* = 13.35; Figure 1c) among the three groups. Additionally, within all three groups, participants' anxiety and depression scores do not correlate with the brain data from the MOG and SFG regions (all *p* > 0.097).

The further ANOVA results showed that for the right MOG, there were significant differences among the three groups (*F* [2, 50] = 12.84, *p*  < 0.001, partial *η*^2^ = 0.33), with larger GMV of the anxiety (0.41 ± 0.04, *p*=0.031, Cohen's *d* = 1.00, 95%CI [0.012, 0.068]) and the control (0.44 ± 0.03, *p*  < 0.001, Cohen's *d* = 1.70, 95%CI [0.035, 0.085]) groups than the anxiety–depression comorbidity group (0.37 ± 0.04, Figure 1b).

In addition, for the right SFG, there were also significant differences among the three groups (*F* [2, 50] = 9.20, *p*  < 0.001, partial *η*^2^ = 0.27), with a larger GMV of the control group (0.45 ± 0.04, *p*  < 0.001, Cohen's *d* = 1.55, 95%CI [0.038, 0.102]) than the anxiety–depression comorbidity group (0.38 ± 0.05, Figure 1d).

### 3.3. Cortical Thickness Results

**Figure 2:**
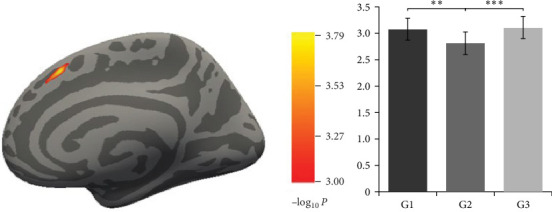
There was a significant difference in the cortical thickness of the (a) right superior frontal gyrus among the three groups and (b) the cortical thickness of the three groups in the right superior frontal gyrus. *Note:* G1 = anxiety group; G2 = anxiety–depression comorbidity group; G3 = control group. *⁣*^*∗∗*^ indicates *p*=0.002 and *⁣*^*∗∗∗*^ indicates *p* < 0.001.

The results showed that there was a significant difference in the cortical thickness of the right SFG ([8.3, 22.1, 47.9], cluster size = 205.56 mm2, −log10*p*=3.78; Figure 2a) among the three groups.

The further ANOVA results showed that for the right SFG, there were significant differences among the three groups (*F* [2, 50] = 9.73, *p*  < 0.001, partial *η*^2^ = 0.30), with larger cortical thickness of the anxiety (3.08 ± 0.21, *p*=0.002, Cohen's *d* = 1.23, 95%CI [0.114, 0.426]) and the control (3.11 ± 0.19, *p*  < 0.001, Cohen's *d* = 1.47, 95%CI [0.154, 0.446]) groups than the anxiety–depression comorbidity group (2.81 ± 0.24, Figure 2b).

### 3.4. Correlation Results Between Hormones and Brain Regions

**Figure 3:**
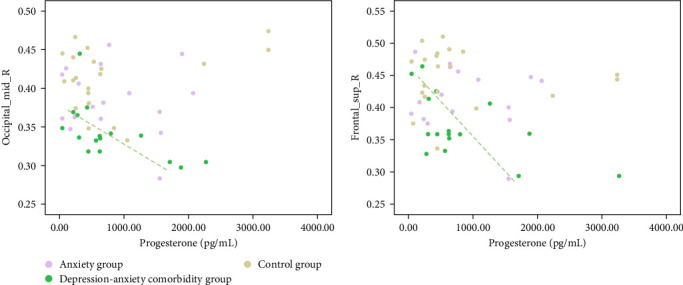
The correlation between progesterone and brain volume.

The brain regions with significant differences in GMV and cortical thickness obtained from the above analysis were selected to test the correlation between individual hormone levels and the extraction value of brain regions in each group. The results showed that the progesterone level was negatively related to the cortical volume of the right MOG (*r* = −0.48, *p*=0.042, Figure 3a) and the right SFG (*r* = −0.53, *p*=0.020, Figure 3b) only in the anxiety–depression comorbidity group. However, the correlation between estrogen levels and the two brain regions was not significant (all *p* > 0.405). Additionally, within all three groups, participants' anxiety and depression scores do not correlate with the estrogen and progesterone levels (all *p* > 0.070).

## 4. Discussion

The present study investigated structural brain alterations and hormone–brain associations in young women with anxiety, CAD, and healthy controls. Consistent with our hypotheses, the CAD group exhibited significantly reduced GMV in the right SFG and right MOG compared to both the anxiety and control groups. In addition, cortical thickness in the right SFG was also significantly lower in the CAD group, supporting the hypothesis that comorbidity is associated with more pronounced structural deficits. Although no significant group differences were found in estradiol or progesterone levels, a significant negative correlation was observed between progesterone and GMV in the right MOG and SFG, specifically in the CAD group. These findings partially support our third hypothesis and suggest that altered progesterone-brain structure associations may be uniquely implicated in the pathophysiology of CAD in young women.

First, the results of our study showed that the cortical volume of the right MOG and the right SFG in the comorbidity group was significantly lower than that in the anxiety group and the control group, with no significant difference found between the anxiety and control groups. This result is consistent with prior evidence showing that reduced GMV in the prefrontal and occipital regions is a common feature among individuals with depressive symptoms but is typically absent in those with anxiety disorders alone [14, 39–42]. Both the SFG and MOG are critical brain regions for attention, executive function, and emotional regulation [43–45]. We propose two mechanistic pathways to explain these structural differences and their role in the transition from anxiety to depression. For one thing, previous studies suggest that individuals with pure anxiety exhibit an increase in GMV, possibly as a compensatory mechanism to help regulate negative emotions [20, 46]. However, this compensatory mechanism appears absent or exhausted in those who transition to CAD, as evidenced by the observed GMV reduction. This suggests that the decrease in GMV may reflect impaired emotional compensation in individuals and could indicate the onset of CAD. For another, individuals with comorbidities exhibit more pronounced right frontal asymmetry and commit more errors on cognitive tasks [47], suggesting that reduced GMV may predict deficits in cognitive control. This impaired cognitive control specifically affects the ability to disengage from rumination, directly precipitating the emergence of depressive symptoms [48]. This suggests that the decrease in GMV may reflect impaired cognitive control ability and could potentially indicate the onset of CAD. Therefore, the GMVs in the right MOG and SFG of young female anxiety individuals show a decreasing tendency with the onset of depressive symptoms, indicating the worsening of emotional and cognitive disorders.

Second, the results also showed that the cortical thickness of the right SFG of the anxiety–depression comorbidity group was significantly lower than that of the control group and the anxiety group. No differences were found between the anxiety group and the control group. The prefrontal cortex edge, especially the orbitofrontal cortex, plays a critical role in decision-making and emotion regulation [49]. Previous studies have also confirmed that cortical thickness was reduced in the parietal, lateral prefrontal cortex, and orbital frontal cortex in patients with depressive symptoms, which indicates worse emotional regulation [50]; however, these were not found in patients with anxiety. Interestingly, in comparison to individuals with simple anxiety or depression disorders, research has revealed a further reduction in the cortical thickness of the SFG among patients with CAD [51]. As it turned out, the additional atrophic regions in these patients involve key nodes in the anatomical circuit of anxiety [50]. This suggests that the combination of anxiety and depression leads to a thinning of cortical thickness. Therefore, we believe that the adjustment of prefrontal cortical thickness, especially in the SFG, is the foundation and key to intervening in patients with CAD. Further research indicates that multidomain cognitive and motor training is associated with significant increases in the volume and thickness of the frontal cortex, thereby enhancing individuals' attention, emotion, and memory functions [52–55]. Therefore, developing appropriate cognitive and motor training programs is meaningful for improving emotional symptoms in women with CAD.

In addition, our study analyzed ovarian hormone levels across three groups, revealing a negative correlation between progesterone levels and the GMV of the right MOG and the right SFG within the anxiety–depression comorbidity group. We speculate that progesterone plays a role in the reduction of brain volume in women with comorbidities. Consistent with our results, previous studies show that in individuals with typical menstrual cycles, no associations are observed between estradiol and changes in brain structure. In contrast, increased progesterone levels are associated with fluctuations in brain volume, characterized by reductions in the volume of regions related to the frontal and temporal lobes [56, 57]. Importantly, this negative correlation was not found in our healthy or anxiety-only groups. Prior research has shown that in depressive-anxiety comorbid conditions such as premenstrual dysphoric disorder (PMDD), the brain is more sensitive to hormonal fluctuations like those of progesterone [58]. This increased sensitivity may lead to structural changes in the brain. These findings support the group-specific effects seen in our study. Furthermore, there was no statistically significant difference in hormone levels across the three groups, but a discernible pattern emerged with progressively higher progesterone observed among the control group, anxiety group, and anxiety–depression comorbidity group, correlated with the accumulation of emotional symptoms. Studies suggest that elevated progesterone levels during the luteal phase are positively correlated with emotional reactivity in the dorsolateral prefrontal cortex of women, which may contribute to the exacerbation of mood disorders [59, 60]. Therefore, we speculated that there was no significant difference in hormone levels among the three groups, which might be attributed to the limited sample size and the infrequent collection of saliva during the menstrual cycle. However, further research indicates that progesterone has both anxiolytic and anxiogenic effects, mediated by its metabolites pregnenolone and allopregnanolone [61]. Consequently, the relationship between progesterone and female mood disorders depends on the timing of measurement and the influence of these metabolites. Given the current state of research, progesterone should still be used cautiously as a biomarker for the worsening of female mood disorders.

## 5. Limitation

Some limitations must be noted. First, we could not find young women who had been with anxiety at first and then developed into CAD; thus, this study only divided the subjects into the anxiety group and the anxiety–depression comorbidity group to distinguish the difference between the two groups. Future research can be carried out to pay attention to the development and changes of women with anxiety disorders. Second, the sample size of subjects was limited. According to our data, most young women had both anxiety and depression, making it hard to find those with only depression. We speculated that since the participants were students from four relatively good universities, compared with their peers, they felt more pressure, which led to a generally higher level of anxiety. Future research should expand the number of participants. Although large effect sizes and narrow 95% confidence intervals were observed in our key findings, the small sample size still calls for cautious interpretation. Future studies should recruit more diverse participants and increase the sample size to enhance statistical power and the robustness of the results. Third, in this study, only the premenstrual period of the menstrual cycle was selected to measure the hormone level, but the hormone levels of the menstrual phase and the luteal phase were not measured. Future studies should measure changes in hormones throughout the cycle to obtain more comprehensive results. Finally, although we have adopted a ComBat technique to reduce the scanner effect, inconsistent scanning parameters during data collection between the two centers may also have some potential impact on the results.

## 6. Conclusion

Overall, our study confirmed that reduced GMV and cortical thickness in the right SFG and MOG may underlie the progression from anxiety to CAD in young women. Moreover, the observed negative correlation between progesterone levels and brain structure, specifically in the comorbidity group, suggests a potential hormone-related mechanism contributing to these structural alterations. These findings highlight the joint role of neuroanatomical changes and hormonal factors in the pathophysiology of emotional comorbidity and underscore the value of integrating neuroimaging and endocrine markers to inform early identification and intervention strategies in this population.

## Figures and Tables

**Table 1:** Demographic variables (M ± S D).

Variables	Anxiety group	Anxiety–depression comorbidity group	Control group	*F*	*p*-Value^a^
Age	20.95 ± 2.09	22.17 ± 2.20	22.10 ± 1.63	2.49	0.093
BMI	19.84 ± 2.28	20.58 ± 1.76	20.53 ± 2.09	1.40	0.255
BAI	43.42 ± 7.28	45.89 ± 6.48	25.88 ± 2.13	69.13	<0.001
BDI	6.26 ± 3.51	21.44 ± 3.75	1.10 ± 1.15	171.52	<0.001
Progesterone (pg/mL)	814.68 ± 679.38	898.81 ± 1038.64	795.59 ± 959.93	0.01	0.898
Estradiol (pg/mL)	35.81 ± 33.83	21.69 ± 17.26	21.38 ± 20.52	1.94	0.154

*Note*: BMI is the body mass index, which was calculated by dividing the weight (kg) by the height (m) squared (kg/m^2^).

^a^The *p*-value was obtained from the outcomes of comparison between the three groups.

## Data Availability

The data that support the findings of this study are available from the corresponding author upon reasonable request.
